# The prevalence of anxiety and depression in bronchiectasis patients and their association with disease severity: a cross-sectional study

**DOI:** 10.1038/s41598-023-48276-1

**Published:** 2023-11-28

**Authors:** Khaled Al Oweidat, Dana Marie, Ahmad A. Toubasi, Dunia Z. Jaber, Khalid E. Ahmed, Bayan O. Abu Alragheb, Asma S. Albtoosh

**Affiliations:** 1 Department of Respiratory and Sleep Medicine, Department of Internal Medicine, School of Medicine, The University of Jordan, Amman, Jordan; 2 Faculty of Medicine, School of Medicine, The University of Jordan, Amman, Jordan

**Keywords:** Psychology, Diseases

## Abstract

Bronchiectasis is a chronic lung disease characterized by recurrent respiratory symptoms. Several studies demonstrated that psychological comorbidities are common in patients with bronchiectasis. The aim of this study is to investigate the prevalence of anxiety and depression in bronchiectasis patients and assess their association with disease severity. In this cross-sectional study, we included patients diagnosed with bronchiectasis. The study was conducted using an interviewer-administered questionnaire via phone calls and data collected from the electronic medical records at JUH. The questionnaire included patients’ demographics and disease characteristics. Anxiety and depression were assessed using GAD7 and PHQ9 respectively. Bronchiectasis disease severity was assessed using BSI and FACED score. The total number of included patients was 133. Moreover, 53.4% of the participants were females while the rest were males (46.6%). PHQ9 demonstrated that 65.4% of the patients had depression. Regarding anxiety, GAD7 scale showed that 54.1% of the patients had anxiety. Pearson correlation showed that bronchiectasis severity index was significantly associated only with PHQ9 depression scores (r = 0.212, *P* value = 0.014). The prevalence of depression and anxiety is high among patients with bronchiectasis. We believe that patients affected with bronchiectasis should be screened for depression to improve their quality of life.

## Introduction

Bronchiectasis is a chronic suppurative lung disease that is characterized by permanent bronchial dilatation and recurrent respiratory symptoms. Bronchiectasis could be a debilitating disease and often takes a negative toll on the patient’s quality of life, which could result in the patient developing psychological sequels. It is established in the literature that there is a higher incidence of psychological co-morbidities in patients with chronic lung diseases as opposed to the general population, and bronchiectasis is no exception to that^1,2^. However, the link between the severity of bronchiectasis symptoms and the incidence of depression and anxiety is an area of conflict in the literature.

In 2021, a Korean multi-center cohort study examined the correlation between the prevalence of depression and disease severity in 810 bronchiectasis patients. They used the PHQ-9 scale to assess depressive symptoms and found 20.7% of patients had relevant depression while only 11.9% had a diagnosis of depression, they also found a significant association between having depression and the severity of the disease which was measured by the bronchiectasis severity index (BSI) and E-FACED^3^. A cross-sectional study from Turkey found that of 90 bronchiectasis patients recruited, 30% were diagnosed with anxiety while 41% were diagnosed with depression, with female participants having significantly higher incidences. The study also measured disease severity using the BSI and FACED severity indices but failed to find any significant correlation with anxiety or depression^4^. Likewise, a 2018 cross-sectional Chinese study including 163 bronchiectasis patients found a high rate of depression (30%) and anxiety (40%) as opposed to healthy subjects (10% and 6.1% respectively), but it also failed to establish a link between incidence and disease severity when using the BSI and FACED scores^5^.

Large scale studies examining the link between bronchiectasis severity and anxiety or depression’s prevalence are both scarce and conflicting in findings. In addition, no study has been previously conducted in the Middle East region, which just like any ethnic region its inhabitants have a unique attitude and reactions towards illness that should be reported. The aim of this cross-sectional Jordan based study is to measure the prevalence of anxiety and depression among participants, as well as exploring their association with bronchiectasis severity.

## Methods

### Study design and setting

This study is a retrospective cross-sectional study, conducted using an interviewer-administered questionnaire via phone calls. Our target population was patients diagnosed with bronchiectasis who were admitted to the respiratory department at Jordan University Hospital between 2012 and 2022. We called the patients and asked them for answers to our questionnaire. Also, we extracted relevant data from electronic medical records (%FEV1, pseudomonas colonization, colonization with other organisms, and radiologic severity). We recruited responses from the 10th of January 2023 to the 10th of February 2023.

### Population

A total of 368 bronchiectasis patients were admitted between 2012 and 2022, but only 133 responded and were willing to participate. Patients were included if they met the inclusion criteria: if the patient’s age was 18 or older, diagnosed with bronchiectasis by high-resolution CT scan, and had no exacerbations in the last 4 weeks. We considered exacerbation as a worsening of symptoms that led to hospital admission. An exacerbation assessment was done by taking history and reviewing medical records. The estimated prevalence of depression in the Jordanian population according to the literature was 13.3% while the prevalence of anxiety was 23.7%^6,7^.

### Questionnaire

The questionnaire was composed of three sections; the demographics part, followed by four validated scoring systems to measure our three outcomes of interest (bronchiectasis severity, anxiety, and depression)^8–11^. We used the bronchiectasis severity index (BSI) and the FACED score for evaluating bronchiectasis severity. BSI uses the following criteria: (BMI, predicted value of forced expiratory volume in the first second (%FEV1), number of admissions and exacerbations, MMRC breathlessness score, pseudomonas colonization, colonization with other organisms, and radiologic severity). Based on BSI, bronchiectasis severity is described as mild (0–4), moderate (5–8), or severe (9 or more). The FACED score consists of five dichotomous variables (%FEV1, Age, Chronic colonization, Extension, Dyspnea). Its score was categorized into mild (0–2), moderate (3–4), or severe (5–7). In addition, the Arabic version of the Patient Health Questionnaire (PHQ-9) was used to assess nine depression criteria. While generalized anxiety disorder severity was measured using a seven-item Generalized Anxiety Disorder Assessment (GAD-7) questionnaire. In PHQ-9 and GAD-7, Each Item was given a score of “0” (not at all) to “3” (nearly every day).

### Ethical approval

This study was authorized by the International Review Board (IRB) Committee of the University of Jordan Hospital and the faculty of medicine. The IRB number was 10/2023/534. Moreover, before completing the questionnaire, a written informed consent was taken from the patient after describing the aims of the study. All the performed methods were conducted in accordance with the relevant guidelines and regulations including the Declaration of Helsinki.

### Data analysis

Categorical variables were presented as counts and percentages while the continuous variables were interpreted as mean, standard deviation and range. The correlations between bronchiectasis severity scores and depression and anxiety scales was tested using Pearson Correlation Coefficient. Chi-square test was used to assess difference in anxiety and depression between patients with other comorbidities and patients without. Any test with a *P* value < 0.05 was considered significant. The data analysis was done using IBM-SPSS v.25.

## Results

### Characteristics of the included patients

The total number of included patients was 133. Moreover, 53.4% of the participants were females while the rest were males (46.6%). The mean age of the participants was 50.43 ± 15.14. Around half of the patients had university-based education. Additionally, 13.5% of the patients were smokers. Only 0.8% of the patients had history of TB and 7.5% of the patients complained of hemoptysis. Furthermore, 75.9% of the patients had diabetes and 34.6% of them had hypertension. Also, 12.8% of the patients had cardiovascular diseases and 6.0% of them had chronic lung disease. The mean disease duration among the patients was 11.68 ± 12.65. Table 1 describes the characteristics of the included patients.

**Table 1:** Characteristics of the included patients.

Variable	Response	Frequency (*n* = 133)	Percentage (%)
Sex	Male	62	46.6
	Female	71	53.4
Education	Primary/secondary	68	51.1
	University	65	48.9
Employment	School	49	36.8
	Employed	37	27.8
	Retired	47	35.3
Smoking status	Non-smoker	86	64.7
	Smoker	18	13.5
	Ex-smoker	29	21.8
Tuberculosis	Yes	1	0.8
	No	132	99.2
Hemoptysis	Yes	10	7.5
	No	123	92.5
Diabetes mellitus	Yes	101	75.9
	No	32	24.1
Chronic kidney disease	Yes	3	2.3
	No	130	97.7
Hypertension	Yes	46	34.6
	No	87	65.4
Autoimmune	Yes	15	11.3
	No	118	88.7
Cardiovascular diseases	Yes	17	12.8
	No	116	87.2
Chronic lung diseases	Yes	8	6.0
	No	125	94.0

Variable	Mean	SD	Range
Age	50.43	15.14	18–77
Disease duration	11.68	12.65	1–60
Bronchiectasis severity index	6.41	3.77	0–16
FACED score	1.90	1.24	0–5
PHQ9 score	8.44	6.86	0–28
GAD7 score	6.10	5.07	0–18

### Bronchiectasis disease severity

**Figure 1:**
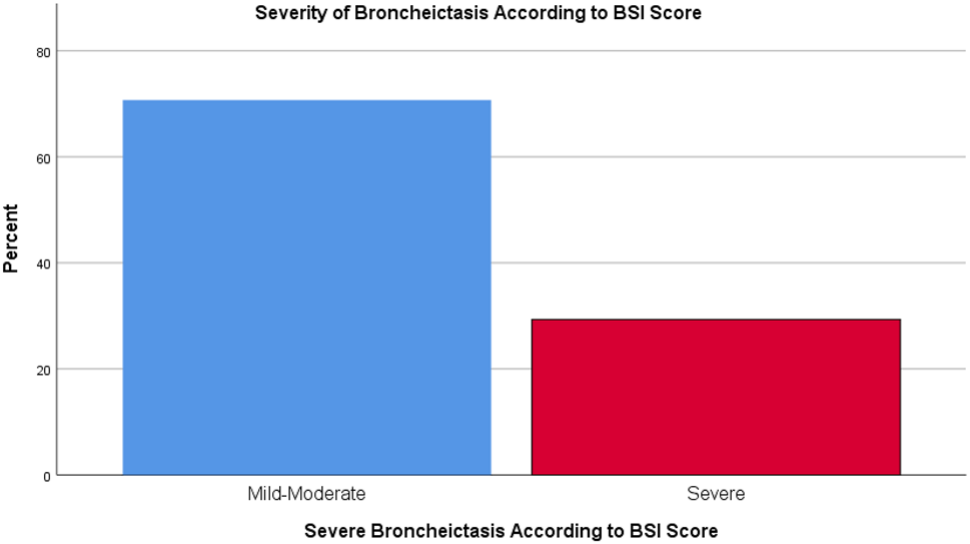
Bronchiectasis severity according to BSI.

**Figure 2:**
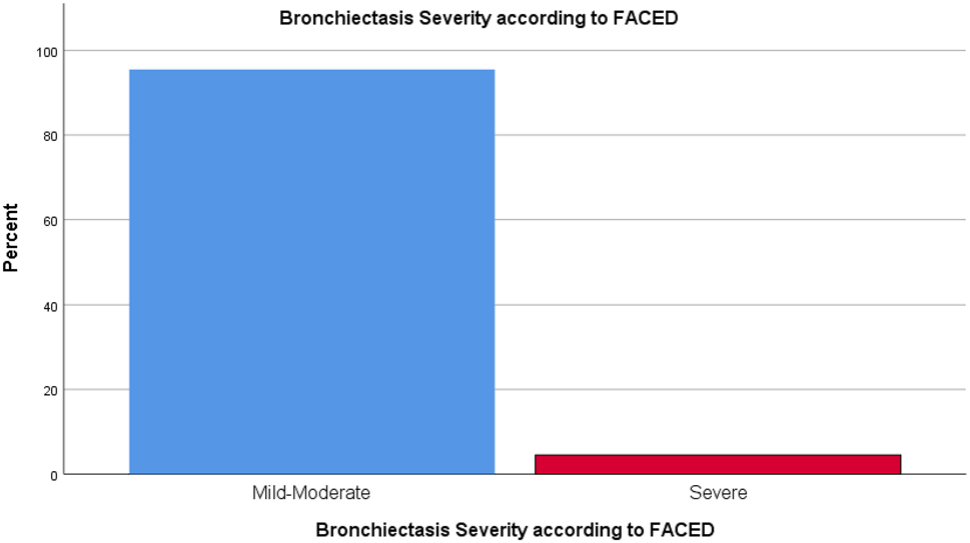
Bronchiectasis severity according to FACED.

According to BSI assessment, 29.3% of the patients had severe bronchiectasis (Fig. 1). On the other hand, FACED assessment showed that only 4.5% of the patients had severe bronchiectasis (Fig. 2). The mean BSI and FACED scores were 6.41 ± 3.77 and 1.90 ± 1.24, respectively (Table 1).

### Depression and anxiety among bronchiectasis patients

**Figure 3:**
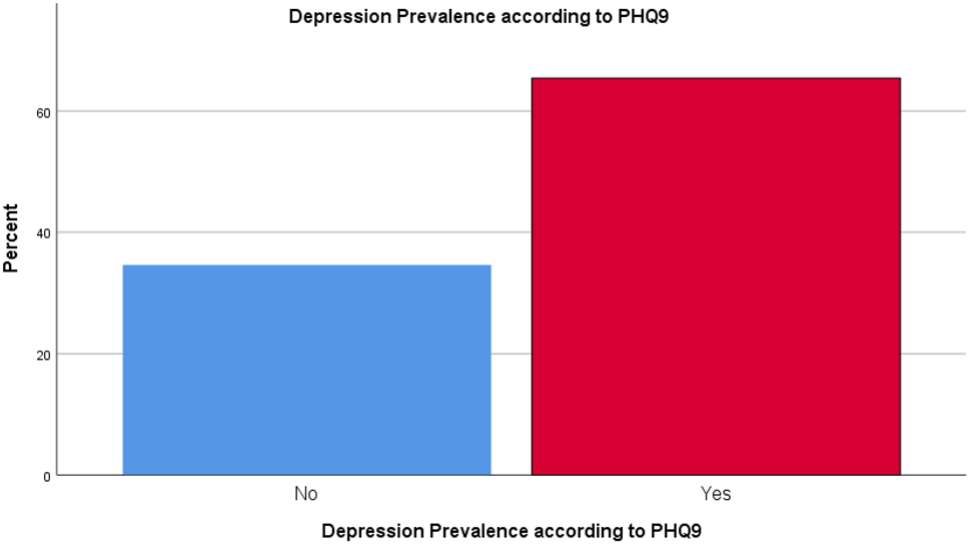
Depression prevalence according to PHQ9.

**Figure 4:**
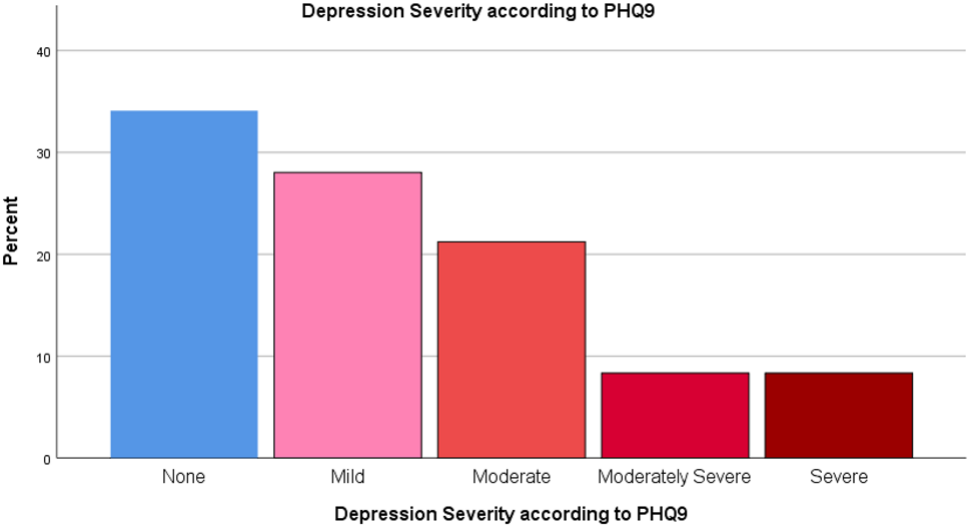
Depression severity according to PHQ9.

**Figure 5:**
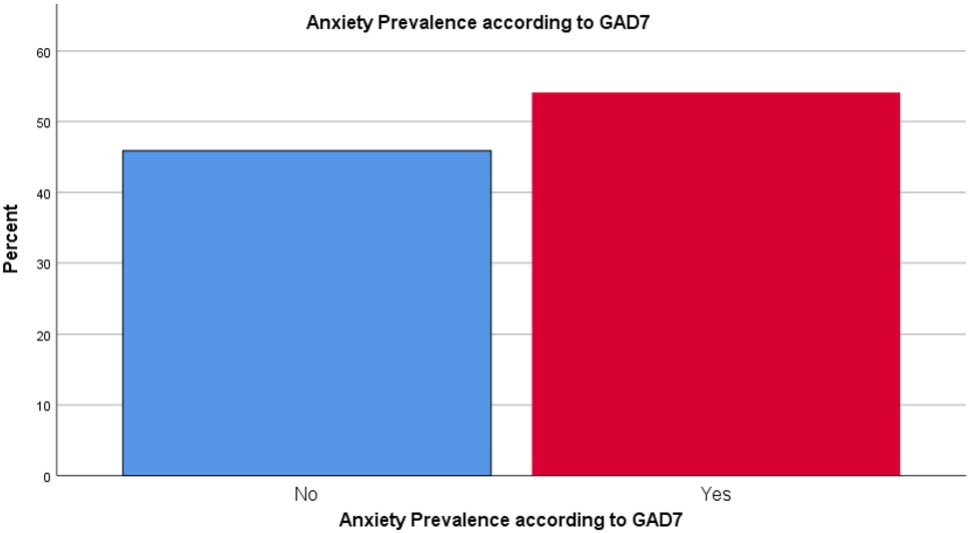
Anxiety prevalence according to GAD7.

**Figure 6:**
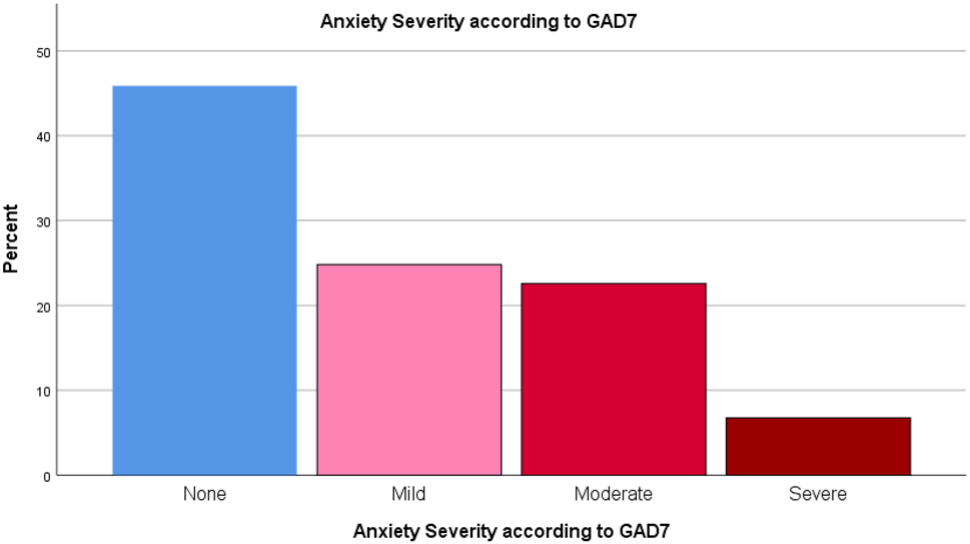
Anxiety severity according to GAD7.

The mean PHQ9 score was 8.44 ± 6.86. PHQ9 demonstrated that 65.4% of the patients had depression (Fig. 3). Of them, 28.0% had mild depression, 21.2% had moderate depression, 8.3% had moderately severe depression and 8.3% had severe depression (Fig. 4). Regarding anxiety, GAD7 scale showed that 54.1% of the patients had anxiety (Fig. 5). Severity scales revealed that 24.8% had mild anxiety, 22.6% had moderate anxiety and 6.8% had severe anxiety (Fig. 6). The mean GAD7 score was 6.10 ± 5.07 (Table 1). Patients with other lung diseases had higher depression and anxiety prevalence compared to patients without other lung diseases but the difference between the two groups was not statistically significant (*P* value > 0.050) (Table 2). In addition, no comorbidity was associated with depression or anxiety (*P* value > 0.050) (Table 3).

**Table 2:** Difference in depression and anxiety prevalence between patients with other lung diseases in addition to bronchiectasis and patients without.

Variable	Response	Patients with lung diseases	Patients without lung diseases	*P* value
Depression	Yes	6 (75.0)	81 (64.8)	0.557
	No	2 (25.0)	44 (35.2)	
Anxiety	Yes	5 (62.5)	67 (53.6)	0.624
	No	3 (37.5)	58 (46.4)

**Table 3:** Investigating the comorbidities associated with depression.

Variable	Response	Depression		*P* value	Anxiety		*P* value
		Yes	No		Yes	No	
Diabetes	Yes	20 (23.0)	12 (26.1)	0.691	16 (22.3)	16 (25.9)	0.982
	No	67 (77.0)	34 (73.9)		45 (77.7)	56 (74.1)	
Chronic kidney disease	Yes	1 (1.1)	2 (4.3)	0.237	71 (96.7)	59 (98.6)	0.465
	No	86 (98.9)	44 (95.7)		1 (1.4)	2 (3.3)	
Hypertension	Yes	56 (64.4)	31 (67.4)	0.727	22 (30.6)	24 (39.3)	0.288
	No	31 (35.6)	15 (32.6)		50 (69.4)	37 (60.7)	
Autoimmune diseases	Yes	10 (11.5)	5 (10.9)	0.914	8 (11.1)	7 (11.5)	0.947
	No	77 (88.5)	41 (89.1)		64 (88.9)	54 (88.5)	
Coronary artery disease	Yes	10 (11.5)	7 (15.2)	0.541	9 (12.5)	7 (11.5)	
	No	77 (88.5)	39 (84.8)		63 (87.5)	54 (88.5)	

### Correlations between bronchiectasis severity scales and anxiety and depression scales

Pearson correlation showed that bronchiectasis severity index was significantly associated only with PHQ9 depression scores (r = 0.212, *P* value = 0.014). No significant correlation was found between FACED score and PHQ9 depression score or GAD7 anxiety score (*P* value > 0.0) (Table 4).

**Table 4:** Correlations between bronchiectasis severity scales and anxiety and depression scales.

Variable	PHQ 9 scale		GAD7 scale	
	Correlation coefficient (r)	*P* value	Correlation coefficient (r)	*P* value
Bronchiectasis severity index	0.212	0.014*	0.030	0.735
Faced score	0.046	0.596	0.006	0.942

*P value < 0.05.

## Discussion

Anxiety and depression are common comorbidities in patients with chronic diseases including chronic airway lung diseases^12^. Notably, anxiety and depressive symptoms are often overlooked by physicians and have been associated with worse adherence to treatment and overall increased morbidity in patients with chronic airway lung diseases^13–15^.

The findings in this study are consistent with the findings of previous studies done on this topic that anxiety and depression are common among patients with bronchiectasis. Our study demonstrated that the prevalence of depression in bronchiectasis patients is 65.4% and that of anxiety is 54.1%. However, those numbers are higher than those reported by other studies. Olveira et al.^1^ reported a prevalence of 20% and 38% for depression and anxiety, respectively. On the other hand, a Korean study done on outpatients with chronic airway lung diseases had a depression prevalence of 55% in patients with bronchiectasis, similar to that of COPD patients, and anxiety prevalence of 39% which was higher than that in patients with COPD or asthma^16^. A Turkish cross-sectional study, which only included non-cystic fibrosis bronchiectasis patients, reported a prevalence of 41% and 30% for depression and anxiety, respectively^4^. A study by Girón Moreno et al.^17^ conducted in Spain reported a depression rate of 34% among non-cystic fibrosis bronchiectasis patients and 55% for anxiety.

The discordance in the prevalence rates of anxiety and depression may be due to differences in patients’ characteristics, differences in methodologies and the use of different screening questionnaires for depression and anxiety. Patients in this study had higher mean age than some previous studies, the aforementioned Olveira et al. study presented increasing rates of depression and anxiety in relation to increasing age. Although the questionnaires used to assess the psychological status are validated screening tools, they might overestimate the rates of anxiety and depression^18^. Furthermore, bronchiectasis patients frequently suffer from fatigue, sleep disturbances and reduced appetite which are incorporated into the screening tools for psychological status may have consequently resulted in falsely elevated rates of depression and anxiety. Approximately 75.9% of the participants in this study have diabetes which has been linked to high prevalence of comorbid depression and anxiety^19,20^. However, there may also be social factors contributing to the high prevalence of anxiety and depression in patients in this study compared to the studies from other countries.

Bekir et al.^4^ reported no association between anxiety and depression rates and bronchiectasis disease severity assessed with BSI and FACED. This is consistent with the Chinese cross-sectional study where they suggested that anxiety and depression in bronchiectasis patients might have originated from other mechanisms unrelated to the disease severity^5^. No significant association was found between anxiety and disease severity scores (BSI and FACED) in the present study. However, this study demonstrated that depression scores was associated with disease severity only when assessed by BSI but not FACED. This finding is consistent with the finding of a Korean multicenter cohort which found a significant association between depression, assessed by PHQ9, and disease severity assessed by BSI and EFACED but reported no association with FACED score, hence they suggested exacerbation to be a significant factor associated with depression^3^. The differences in the association between the two scores can be explained by the fact that each of these scores consider different aspects as BSI score includes factors that FACED does not such as BMI, number of exacerbations in the last year and pseudomonas colonization.

This study includes some limitations that should be noted. First, it was conducted in a single tertiary hospital that usually receives severe and advanced patients compared to all affected patients. Second, the small number of patients recruited can be considered as a limitation as well which might impact the generalizability of our findings. In addition to that, the psychological and social status of the patients were examined for the patients’ present status but the development of it over time was not examined. Finally, disease activity was reported by the patients and no specific biomarkers or measures were used to evaluate it.

## Conclusion

This study showed that the prevalence of depression (65.4%) and anxiety (54.1%) is high among patients affected with bronchiectasis. Disease severity index is significantly associated with depression but not anxiety. This associations can lead to much worse clinical consequences that can affect the progress of the disease. We believe that patients affected with bronchiectasis should be screened for depression to improve their quality of life.

## Data Availability

The data associated with this manuscript are available from the corresponding author upon reasonable request.
